# Long-Term Stability in Temporomandibular Joint Replacement: A Review of Related Variables

**DOI:** 10.3390/dj12110372

**Published:** 2024-11-20

**Authors:** Erick Vargas, Victor Ravelo, Majeed Rana, Alejandro Unibazo, Sergio Olate

**Affiliations:** 1 Division of Oral and Maxillofacial Surgery, C.H.M Hospital, Chillán 3810525, Chile; 2 Fellowship in Orthognathic and Complimentary Facial Surgery, Universidad de La Frontera, Temuco 4780000, Chile; 3 PhD Program in Morphological Sciences, Universidad de La Frontera, Temuco 4780000, Chile; 4 Center for Research in Morphology and Surgery (CEMyQ), Universidad de La Frontera, Temuco 4780000, Chile; 5 Department of Oral and Maxillofacial Surgery, Heinrich Heine University Hospital Düsseldorf, 40225 Düsseldorf, Germany; 6 Division of Oral and Maxillofacial Surgery, A.G.P. Hospital, Lautaro 4860133, Chile; 7 Division of Oral, Facial and Maxillofacial Surgery, Universidad de La Frontera, Temuco 4811230, Chile

**Keywords:** TMJ prosthesis, TMJ replacement, facial deformity, TMJ

## Abstract

**Background:** The temporomandibular joint (TMJ) is a key component of the stomatognathic system, playing a major role in maintaining mandibular stability and function. Temporomandibular disorders (TMDs) are a prevalent disease in the world, with surgical treatment being reserved for complex cases or end-stage TMJ disease. A narrative review was conducted to describe the quantitative and qualitative factors that affect TMJ prosthesis stability. **Methods:** Studies with a sample size equal to or greater than 10 subjects who underwent surgical procedures for joint replacement using stock or customized ATM prostheses were included. This narrative review examined some variables that may influence in terms of the longevity of the TMJ prosthesis, highlighting issues to be considered in future research. **Results:** The current development of TMJ prostheses is benefiting from technological advances, offering a suitable adaptation to the patient’s anatomy and superior results in functionality and patient satisfaction. However, the biomechanical complexity of the TMJ shows unique challenges compared to other joints in the body, where anatomical, biomechanical, and functional requirements are high. The stability of the TMJ prosthesis is affected by multiple variables, including the selection of biocompatible materials that resist corrosion and wear, the design of the prosthesis, the diagnosis and indication for its use, and the surgeon’s experience. The success of TMJ replacement can be measured by improving the patient’s quality of life, reducing pain, restoring mandibular functionality, and recovering suitable facial morphology for the patient’s conditions. **Conclusion:** There is a need for training of maxillofacial surgeons in TMJ surgery and replacement, as well as a greater focus on the research and development of systems to simplify surgical design and procedures and to optimize the results of TMJ replacement.

## 1. Introduction

The temporomandibular joint (TMJ) is a complex structure of the stomatognathic system related to vital functions such as breathing, chewing, and swallowing [1]. The TMJ can present diseases or conditions such as ankylosis, degenerative pathologies, tumors, or others, which can be associated with pain, restriction in mandibular mobility, changes in facial morphology, and a decrease in the patient’s quality of life [2,3]. The prevalence of temporomandibular disorders (TMDs) varies widely among different studies, with estimates ranging from 9.8% to 74% and affecting more women than men [4]. About 90% of joint pathologies respond favorably to conservative or non-invasive treatments so surgical treatment is used in a small group of the population [5]. The main indication for TMJ replacement is often the treatment for severe degenerative disorders and reconstructive needs of the joint, including situations such as osteoarthritis, ankylosis, tumors, and other conditions that result in morphological defects and functional impairment of the TMJ [6]. TMJ replacement with alloplastic systems results from significant technological and scientific advancement, delivering a viable solution for various severe TMJ conditions, and which has been particularly beneficial in cases of severe anatomical discrepancies [7].

The average age for TMJ replacement is wide, reflecting a diversity of pathological conditions. Replacement is usually performed in young patients, with an average age of approximately 40 years [8], in contrast to other joints, such as the knee, where the average age for replacement is over 65 [9]. On the other hand, it has been observed that the design of the prosthesis and the selection of materials for its manufacture affect the biomechanical response of these systems under different loads [10]. The virtual planning technique has improved outcomes and stability, and some reports suggest success rates over 15 years for customized TMJ replacement systems [11].

Successful surgery for TMJ replacement is associated with improved quality of life, reduced pain intensity, improved chewing efficiency, and mandibular mobility in patients with severe degenerative diseases [12]. After alloplastic total TMJ replacement, patients’ perceptions of pain relief, chewing, and quality of life have shown good results [13]. This review aims to analyze the variables associated with the long-term success of prostheses used for total TMJ replacement and to discuss the needs in future developments in the field.

## 2. Materials and Methods

A narrative review was conducted to describe the variables that affect TMJ prosthesis stability in the long term. Studies with a sample size equal to or greater than 10 subjects who underwent surgical procedures for joint replacement using stock or customized TMJ prostheses were included to evaluate clinical conditions. A historical analysis was carried out in terms of variables related to the manufacturing material of the joint prostheses, the biomechanics, the design of the prosthesis, and other variables associated with the surgical or post-surgical procedure. Case reports and animal studies were excluded. The complete list of articles identified in the metasearch engines was imported into the Mendeley 2.90.0 software (Reference Management, Elsevier, London, England). Duplicates were automatically eliminated, and two authors selected the studies related to the variables analyzed. In case of discrepancies, a third author was consulted. The nature of this research is a narrative review, using inclusion/exclusion criteria for clinical articles.

## 3. Historical Background of the TMJ Prosthesis

The first verified scientific papers on TMJ prostheses in patients were produced in the 1960s, using different materials. These prostheses were rudimentary and did not offer long-term solutions for patients with severe TMJ dysfunction. However, they marked a turning point for future innovations [14]. During the 1980s, partial implants such as the Proplast–Teflon and the total joint replacement system (Vitek Inc., Houston, TX, USA) were widely used. These promised better integration with human tissue and a low rate of secondary reactions [15]. However, in the medium- and long-term follow-up, it was observed that Proplast–Teflon disintegrated and caused foreign body reactions and severe inflammatory reactions, which led to its withdrawal from the market in the 1990s [16].

**Figure 1:**
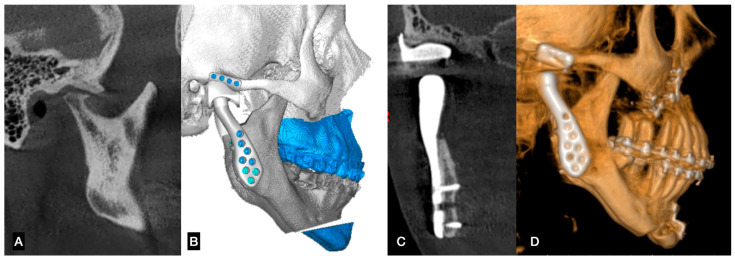
(**A**) End-stage TMJ disease, (**B**) TMJ replacement planning using a personalized system, (**C**) postoperative results showing a good fit between fossa and condyle component, (**D**) TMJ replacement with orthognathic surgery in the same procedure. (Author’s case, TMJ prosthesis by Artfix Implants (Pinhais—PR, Brazil)).

At the end of the 20th century, with the introduction of CAD/CAM technology in maxillofacial surgery, there was a significant development in surgical planning and the manufacture of osteosynthesis systems [8] (Figure 1). This enabled the creation of customized implants in different areas of surgery, including the TMJ prosthetic system. Materials used during this era included titanium and ultra-high-molecular-weight polyethylene, known for their durability and biocompatibility with the human body [14].

TMJ prostheses are manufactured using advanced techniques and different methods, offering high customization and adaptability. Today, more than 30 companies worldwide are commercializing these systems. While in the United States (USA), only prostheses from Stryker (formerly TMJ Concepts) and Zimmer Biomet have FDA approval, there are 14 other countries with companies engaged in the planning and manufacture of customized or standard systems for total TMJ replacement [17].

### Temporomandibular Joint and Other Joints

**Figure 2:**
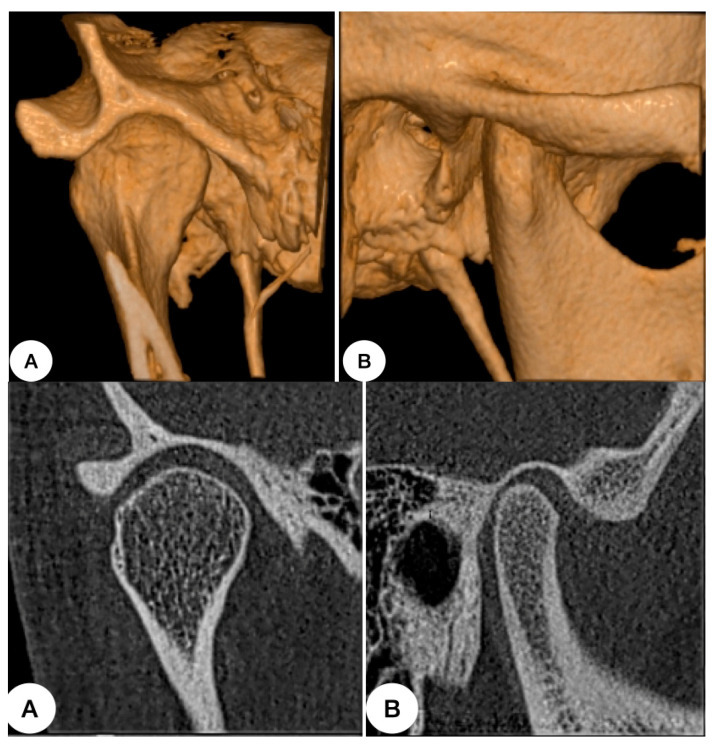
Author’s case: healthy temporomandibular joint. (**A**) Frontal section. (**B**) Sagittal section. Position in the fossa, bone volume, density, morphology, and corticalization is observed in those images.

The TMJ is a complex joint system, sensitive to changes in masticatory loads and occlusal conditions that can influence the development of temporomandibular disorders [18]. Knowing the biomechanical properties of the components is key to understanding the impact of mastication and occlusal forces on the joint [19]. The articular disk is a structure that plays an important role in maintaining the function and health of the TMJ, helping to coordinate the action between different articular surfaces with load distribution during function [20] (Figure 2).

The joint capsule is a fibrous structure that surrounds the joint, providing structural support and containment of the synovial fluid that lubricates the articular surfaces. This capsule and the ligaments help to maintain the integrity and alignment of the joint during opening, closing, protrusion, retrusion, and lateral movements [21]. The temporalis, masseter, and pterygoid muscles work together with the ligaments and joint capsule to facilitate mandibular movements and ensure proper TMJ function [22].

The hip and knee joints were forerunners in the evolution of joint prosthetics, enabling the exchange of expertise to develop TMJ prosthetics [23]. The knee and TMJ are synovial joints with similar structures for stability, such as the articular disk in the TMJ and the meniscus in the knee; however, their function and location are totally different. In terms of biomechanics, the knee bears the weight of the body. It undergoes high compressive and tensile forces during physical activity (3080–3600 N), while the TMJ experiences forces during chewing and other oral functions (770–900 N) that are lower [24].

The incidence of osteoarthritis in the TMJ varies between 28 and 38% [25], in the knee 22.9%, and in the hip 7.4%. However, by 2050, knee osteoarthritis cases are expected to increase by 74.9% and by 78.6% in the hip [26]. Although there are no real projections for the increase in TMJ disorders, it is clear that there is an increase in the consultation and diagnosis of mandibular joint diseases. By 2030, 1,921,000 knee joint replacements are expected to be performed in the US [27], while 902 are projected for TMJ [28]. The number of TMJ replacements is significantly low compared to the knee; one of the reasons for this difference lies in the theoretical and practical training in joint surgery of maxillofacial surgeons (MFSs), which is lower than that observed with orthopedic surgeons (OSs). Some studies indicate that of all procedures performed by MFSs in their last year of training, joint surgery accounted for 3.5%, which were mostly closed treatments (infiltrations), versus 16% for OS24. In addition, many OSs undertake a 1-year post-residency fellowship to obtain specific knowledge, skills, and abilities, which is related to the higher number of orthopedic surgeons trained for joint replacement surgery [29].

Another aspect that influences this difference between TMJ and knee replacement is the disability contributed by alterations to the joint, where the knee disease can generate disability and reduced mobility and displacement in patients [30]. In contrast, damage to the TMJ generates adaptive changes in diet, nutrition, and mandible mobility that patients could maintain for some time [31]. Another significant distinction lies in the financial commitment allocated to the research and development of these systems. Knee prosthetics have had considerable market growth due to the interest of both public and private health systems and the companies involved in their production [24].

Despite these differences, the long-term stability and function of TMJ prostheses have been particularly efficient; it has been reported that the revision rate of a TMJ prosthesis is less than 2% [32], which is significantly lower than other joint prostheses in the human body [33].

## 4. Variables Influencing the Long-Term Stability of TMJ Prostheses

The longevity of a TMJ prosthesis can be influenced by several factors related to the patient, prosthesis design, surgical procedure, and follow-up.

### 4.1. Prosthesis Materials

It is important to consider that the response to the materials currently used in joint prostheses has biological compatibility. Some criteria that must be fulfilled by materials to manufacture joint prostheses include resistance to corrosion, biocompatibility, low wear and fatigue, adaptability to anatomical structures, functional compatibility, and others [34]. In terms of material, a clear example from the past is the Vitek–Kent prosthesis, which had to be withdrawn from the market in the 1990s for generating a marked foreign body reaction and giant cell reaction [14]. It has been observed that increased cobalt levels in the body, especially from metal-on-metal hip prostheses, can lead to severe systemic complications [35]. High rates of chromium and cobalt have also been reported in the body of patients using metal-on-metal TMJ prostheses [36]. Current prostheses composed of ultra-high-density polyethylene and alloys containing titanium, chromium, cobalt, and molybdenum in some of their components are safe with low long-term complication rates [37].

However, hypersensitivity reactions can be triggered by various factors, including the materials used to manufacture the prosthesis. In particular, metal alloys used in the condylar head should consider allergies in patients undergoing total joint replacement procedures [38]. Components such as nickel and chromium in some prosthetic materials can cause adverse reactions and implant failure [39].

Between 10% and 15% of the population has been reported to be metal-sensitive, with nickel allergy being the most common, followed by cobalt and chromium [40]. The prevalence of cobalt sensitivity was also found to be approximately 1%, with a significant degree of cross-reactivity between cobalt and nickel [41]. Metal sensitivity, particularly to nickel, chromium, and cobalt, may be influenced by wear or corrosion, as seen in total knee replacement cases. This wear and corrosion can increase sensitivity to these metals [42]. TMJ Concepts and Zimmer Biomet prostheses have nickel values of less than 1%, reducing the potential risk of severe adverse reactions and promoting a low chance of allergic reactions to the TMJ prosthesis [43]. The failure rate of TMJ prostheses due to metal sensitivity could be 0.33% [44].

### 4.2. Prosthesis Design

Customized prosthetics are notable for allowing a precise adaptation to the patient’s anatomy, which is achieved through advanced technologies such as computer-aided design and 3D printing [45]. These tools facilitate the creation of specific cutting guides and prosthetic components that are optimally matched to individual needs, resulting in shorter operating times and improved structural alignment that can contribute to faster recovery and lower intervention trauma [8].

Despite the obvious advantages of these systems, the main disadvantages are the high cost and longer manufacture times than stock prostheses. These factors may limit accessibility for some patients and surgeons, making standard prostheses still a viable option in many situations [46].

**Figure 3:**
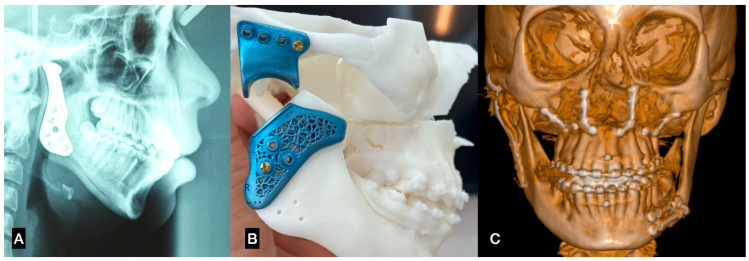
(**A**) Stock TMJ replacement system; in surgery, can be used as a template to choose the best size for the surgery, (**B**) surgical guide used in patient-specific implant (PSI) for TMJ replacement, (**C**) skeletal symmetry obtained in orthognathic surgery and TMJ replacement using PSI.

Recent studies showed statistically that differences in clinical results comparing customized and stock prostheses are not significant in function and stability; their main difference is related to surgical planning and results [46,47] (Figure 3). However, highly complex cases such as larger volume resections of the mandibular ramus and body and the complementary use of other craniomaxillofacial osteotomies have poor analysis in defining the performance of customized prostheses compared to standard prostheses, and the trend in the scientific literature is to use customized prostheses in complex cases.

### 4.3. Surgical Conditions

Experienced surgeons play a critical role in achieving successful patient outcomes. Studies have shown that experienced surgeons exhibit superior technical skills, accuracy, and efficiency in performing joint replacement procedures, leading to reduced operating times, greater surgical precision, and lower complication rates compared to novice surgeons [48,49]. A surgeon’s experience enables them to navigate complex surgical scenarios effectively, improving patient satisfaction and outcomes [50]. Studies have indicated that young surgeons may take longer to complete procedures, show less precision in surgical maneuvers, and have higher rates of errors or complications than experienced surgeons [48,51]. For this reason, the survival of total TMJ replacements is influenced by several factors, including the surgeon’s skill and experience [52]. The results of a study on knee joint replacement highlight that surgeries performed by surgeons with fellowship training in arthroplasty significantly improve the likelihood of treatment success, which is about experience and surgical technique [53]. Another study of hip joint replacement notes the importance of integrating patient preferences with surgeon experience in joint replacement cases. This suggests that experienced surgeons who can align patient needs with clinical expertise are essential to achieving positive outcomes in joint replacement procedures [54].

### 4.4. Biomechanics

In the field of TMJ prosthetics, various biomechanical evaluations have been performed to assess the outcomes of total TMJ replacement with different types of prostheses. The most studied are the customized ones, where their fit and precision are evaluated for each patient, seeking to improve the success and functionality of TMJ replacement procedures [55]. The precision in these systems indicates that a failure in the implant fixation can cause micro-movements, which would favor implant failure due to instability and/or the production of wear debris [56]. On the other hand, the change in biomechanics could be related to differences in responses to stress; a study of using computational models showed that the anterior and posterior temporalis muscle fibers on the side undergoing joint replacement generate less force, resulting in muscle asymmetry [57].

**Figure 4:**
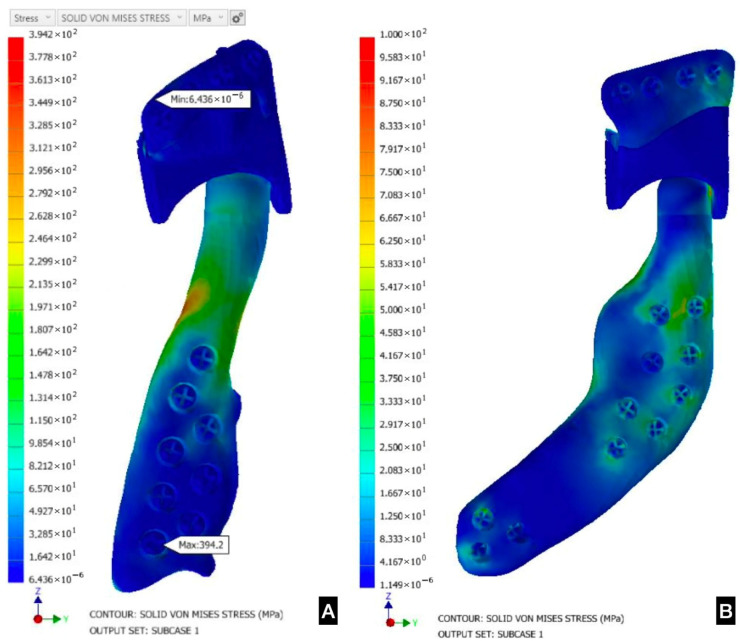
(**A**) Finite element analysis (FEA) in a regular PSI for TMJ replacement showing stress distribution in the upper screw in the condyle component. (**B**) FEA in an extended design showing stress distribution in the posterior screws and in the posterior area of the fossa component.

It has been shown that the use of three staggered screws in the fixation of a TMJ prosthesis can provide optimal stability, stress distribution, and bone tensions [58]. The greatest von Mises stress (this analysis predicts the failure of materials in relation to the load received) is found in the screw holes of the condylar component, areas that are most prone to failure [59] (Figure 4). Customized TMJ prostheses show lower mean stress values in their components and in supporting bone than stock prostheses. Maximum voluntary bite force and oral opening significantly increase in patients with end-stage TMJ pathologies undergoing joint replacement but remain considerably lower than in healthy individuals [60]. Compared to other joints and other prostheses in the body, the biomechanical demand in TMJ prostheses appears to be lower and could significantly influence the long-term success of these systems [23].

Finite element analysis allows defining the parts of a joint prosthesis with the highest stress, as well as evaluating the resistance to elastic modulus and deformation of the material [61]. Vignes et al. [59] observe that the maximum von Mises stress was detected in the screw holes in the condylar components of the implant. Therefore, the screw holes were the areas with the highest probability of failure in the design.

### 4.5. Other Variables

TMJ replacement is a protocolized, defined, stable, and predictable procedure. The indications and criteria should be evaluated by the specialist and the patient, including cases where the indication can be assessed together with other available reconstructive techniques [62]. Hence, the key factors for the success of joint replacements can be determined as follows: the stability of the components, the use of biocompatible materials, the ability to sustain stresses in all ranges of motion, the correct indication, and aseptic installation [1]. Pain experienced by patients with severe joint pathology decreases in most cases after joint replacement; however, in rare cases, pain and discomfort may increase, partially influenced by environmental and psychological factors [63]. Opioid use, previous surgeries, and preoperative pain are predictors of increased postoperative pain [64], suggesting careful evaluation of the indications of earlier surgeries and the use of pain control medications prior to the use of TMJ prostheses.

Young patients undergoing joint replacement and patients with significant comorbidities are at higher risk of prosthetic revision [63]. However, a meta-analysis showed that the percentage of prosthetic revision is low, with heterotopic bone formation and infection being the most common causes [65]. In patients with ankylosis, fat interposition at the time of joint prosthesis installation seems to improve outcomes [66]; therefore, age and initial diagnosis may affect revision requirements.

## 5. Discussion

Currently, the alloplastic TMJ prosthesis is a superior alternative to autologous graft reconstruction in adults; alloplastic replacements in those cases report low complication rates and high predictability, while cases using autologous reconstruction need to maintain the joint immobilization for prolonged periods, a donor site, and the unknow [67,68]. Research based on finite element analysis provides an understanding of biomechanics, defining new designs, and correctly selecting materials [69]. Developing prostheses for other joints has led to a better understanding of biocompatibility and biological response to certain materials [23].

The financial investment in the study of TMJ prostheses is considerably lower than other joints, such as the knee or hip. This could be explained in part by differences in the indication of prostheses in orthopedic surgery versus maxillofacial surgery, the number of surgeons in each area, and the impact that such reconstructions have on the daily life activities of those who suffer from the disease [24]. Understanding the complexity of the TMJ, including its components and movements, is important to continue exploring advancements in prosthetic designs. These enhancements aim to more accurately replicate the joint’s kinematics, approximate the function of a healthy joint, and enhance surgical techniques. The main complications reported are lesions to nerve structures, heterotopic bone formation, and implant infection. Although the rate is close to 2%, it could increase in cases performed by novice surgeons or those with little training in the area [70]. For this reason, it seems necessary to improve the focus on resident training and access to fellowship or sub-specialty programs so that surgeons are prepared to perform these interventions safely and routinely. Another key point to consider is the need for the revision or replacement of the prosthesis in patients with long-term follow-up; considering that TMJ replacements are performed in young patients, long-term stability remains to be understood. For other joints, prosthetic component replacement—such as hip or knee replacement—occurs approximately every 10 to 20 years due to factors like the material biomechanical conditions, installation technique, system stability, and the body’s biological reaction [71,72]. Wolford et al. [73] followed 56 patients undergoing TMJ replacement with TMJ Concepts prostheses over a 21-year period, observing significant improvements in MIO, jaw function, diet, pain, and quality of life. Similar results were obtained in the study by Leandro et al. [74], which carried out a 10-year follow-up of 300 patients undergoing joint replacement with stock prostheses, describing significant improvements in MIO, mandibular function, and decreased pain.

There is a consensus on using TMJ replacement to treat severe conditions such as ankylosis and rheumatoid arthritis. However, for highly prevalent cases like osteoarthritis and arthrosis of the TMJ, there is a lack of consensus, and the procedure may be underused. TMJ prosthesis must be included as a standard component in the treatment of TMJ pathologies.

Based on our review, we observed that digital analysis and 3D manufacturing allow for increasingly precise and personalized prostheses, which allows for a reduction in clinical times [75] and patient safety; patents included recently in this area show the evolution and the success of technology involved in the TMJ prosthesis, showing the impact of recent years for companies involved in this matter.

Joint prosthesis treatment is considered the last technique in therapeutics; therefore, it is necessary to determine a consensus in relation to the diagnosis (clinically and method for diagnosis), since this is the start for the indication of TMJ replacement. Studies [76,77,78] mention that decreased mouth opening, with a liquid or soft diet, loss of mandibular functionality, and pain are the symptoms frequently observed in subjects who are candidates for joint prostheses. These characteristics may be associated with the progressive destruction of the condyle–fossa component, generating facial deformities, so the inclusion of surgeries complementary to the installation of a joint prosthesis will allow the restoration of facial function and aesthetics [77].

In terms of bias and based on our review, we observed that there are no studies with a randomized clinical trial methodology with a sample greater than 10 subjects per group, so this item will be recorded as high-risk bias (based on Cochrane’s bias analyses for clinical trials); furthermore, the inclusion of these patients based on their diagnoses is very specific (most of the patients are included by convenience in the current literature). We also found a lack of blinding by the researchers, since the diagnosis and the surgeons are always experts who are present in both procedures. The main research is American, and the prostheses used in research are mainly related to being FDA-approved. These are hot topics for new research and show a challenge for the coming step in the evolutionary line of TMJ replacement.

## 6. Conclusions

We can conclude that alloplastic TMJ replacement is considered a safe and effective treatment, but the results can be influenced by multiple variables such as the materials, the design, the biomechanics, and others. Several studies mention the importance of the surgeon’s expertise and the lack of studies that evaluate the relationship between long-term clinical results in relation to TMJ replacement. It is necessary to continue developing new technologies and increasing the level of research and training in the area, in order to understand and manage the variables that can influence the success or failure of joint replacements more effectively.

## Data Availability

The data are available upon request from the corresponding author.
